# Barriers to adherence to antiretroviral therapy: identifying priority areas for people with HIV and healthcare professionals

**DOI:** 10.1177/09564624231169329

**Published:** 2023-04-27

**Authors:** Kedar K. V. Mate, Kim Engler, David Lessard, Bertrand Lebouché

**Affiliations:** 1 Department of Family Medicine, 12367 McGill University, Montreal, QC, Canada; 2 Centre for Health Outcomes Research and Evaluation, 507266 Research Institute of the McGill University Health Centre, Montreal, QC, Canada; 3 Chronic and Viral Illness Service, Division of Infectious Disease, 507266 McGill University Health Centre, Montreal, QC, Canada

**Keywords:** Antiretroviral therapy, combination ART (cART), treatment

## Abstract

**Background:**

Challenges to antiretroviral therapy adherence are well-known and continue to be a major hurdle in HIV care. The objective of this paper is to identify barriers to antiretroviral therapy (ART) adherence that are relevant to HIV care from the perspective of people living with HIV and healthcare and social service professionals.

**Methods:**

This study used an online survey design to collect information from the two groups. A total of 100 areas that covered six domains and 20 subdomains were administered to people living with HIV and care professionals in Canada and France. The survey asked participants to rate the importance of each area for HIV care on a four-point Likert scale. Any areas rated 3 or 4 were considered important and ranked. A Chi-square test was conducted for the difference between the groups, people living with HIV and professionals, and between women and men.

**Results:**

A response rate of 87% (58/66) in Canada and 65% (38/58) was achieved. 15 of 43 (35%) areas were endorsed as important barriers by both groups, across countries and sex-covering subdomains — drug cost coverage, challenging material circumstances, HIV stigma, and privacy concerns, affect, motivation, beliefs, acceptance of HIV, comorbidity, side effects, and demands and organisation of daily life. People living with HIV identified two, and care professionals identified nine, additional areas as important barriers to HIV care across different domains and subdomains.

**Conclusion:**

The study identified some common and distinct barriers to ART from the perspective of the people living with HIV and care professionals.

## Introduction

The World Health Organization defines chronic diseases as those conditions that are stable over a long duration or generally very slow to progress.^1–3^ HIV is a prime example thanks to advances in antiretroviral therapy^4,5^ that have been effective in maintaining viral suppression for several decades.^3^ However, maintaining optimal therapeutic drug levels through adequate adherence is critical in managing HIV. Adherence is defined as the extent to which patients follow the recommendations for prescribed treatments.^6^ Non-adherence is associated with a lower CD4 count, increased risk of onward transmission,^7,8^ virus mutation, drug resistance,^9^ AIDS-related mortality,^10–12^ and higher healthcare costs.^13–15^ A review by Machtinger and colleagues reported that a 10% increase in adherence rate is associated with a 28% risk reduction in progression to AIDS (Relative Risk = 0.72; 95% Confidence Interval (95% CI): 0.59–0.87).^12^ Better adherence is shown to be associated with increased success in achieving and maintaining viral suppression, positive health outcomes, and longer life.

The Joint United Nations Programme on HIV/AIDS^16^ proposed an ambitious Fast-Track approach in 2014 with a 95-95-95 strategy to be achieved by 2030 where 95% diagnosed among people living with HIV, 95% on antiviral therapy, and 95% success with viral suppression among those treated.^16^ An additional fourth 90 target for health-related quality of life was recently added.^17^ Over one-third (38%) of people living with HIV have suboptimal adherence to prescribed medication plans, meaning over a third of people do not adhere to the recommended antiretroviral therapy (ART).^18^ In the United States, the adherence rate is reported to be around 74% in a large cohort study of more than 200,000 people living with HIV from 2017 onwards,^19^ which is slightly improved from the 72% adherence rate from 2015 to 2017.^20^ In Canada, the adherence rates among 13,587 people living with HIV studied from 2015–2019, 80% achieved an adherence rate over the target of 90%.^21^

Several factors alone or in combination can affect adherence to medications. These include condition-, patient-, therapy-related factors, health system factors, and social/economic factors. Several reviews suggested patient variables (age, non-white race or ethnicity, sex, lower income, lower literacy, psychological profile, stigma, comorbidities, and unstable housing in resource-rich settings), treatment regimen (complexity and side effects, scheduling issues, number of pills per dose), disease characteristics (HIV-related symptoms), patient-provider relationship (trust in professional, patient’s opinion of the professional’s competence, affective tone and concordance on race or ethnicity), and clinical setting (dedicated adherence program, availability of transportation and childcare, pleasantness of the clinical environment, convenience in scheduling appointments, perceived confidentiality, and satisfaction with past experiences in the health care system) that directly or indirectly affect adherence to antiretrovirals.^22–28^

Non-adherence to ART is a complex, multidimensional, and multifactorial problem that is widely studied, and several strategies are recommended to improve adherence rates in the HIV population. The factors or barriers implicated in non-adherence differ by who is queried and also whether barriers are concordant or discordant between females and male and geographical locations with different healthcare systems. Increasingly literature is focused on the vital influence of patient-provider interactions to ensure the retention of people living with HIV in the healthcare system and improve adherence.^29,30^ However, the barriers to adherence could differ from the perspective of the people living with HIV and healthcare professionals. It is important to consider the differing priorities from the perspective of the groups, and any mediating strategies will be insufficient to target non-adherence to ART.

The patient-provider relationship is key to developing a long-term therapeutic alliance where both parties trust each other and participate equally in shared-decision making to improve health outcomes.^31^ In order to develop any sustainable program to improve adherence rates and empower people living with HIV with strategies to minimize non-adherence, it is critical to identify adherence barriers considered important by people living with HIV and health professionals that can be addressed in HIV care. However, there may not be an agreement on what those are, as perceptions of HIV care priorities as well as of patient adherence and health can differ between these groups.^32,33^

The paper aims to contribute evidence towards people living with HIV and healthcare and social service professionals’ perspectives on the ART adherence barriers that are relevant to HIV care. Specifically, the objective is to estimate the extent to which perceived barriers to ART are endorsed as important to HIV care across sex (female, male), country (Canada, France), people living with HIV, and healthcare professionals. A patient-centered conceptual framework derived from a qualitative synthesis was used to select barriers within each domain and subdomain.^4^

## Methods

### Study design

This study is a cross-sectional analysis of a survey among people living with HIV and healthcare and social service professionals in Canada and France.

### Participants

People living with HIV and healthcare professionals were recruited from four large HIV clinics located in Montreal, St. Jerome, Toronto, and Vancouver in Canada and Paris, Clermont, St. Martin, and Nantes in France. To be eligible, people living with HIV had to be over the age of 18 years and have been taking ART for at least a year. The professionals had to have been practicing for least five years in the HIV field to be eligible for this study. They were recruited through investigators’ research networks and snowball sampling. This group included physicians, nurses, pharmacists, psychologists, psychiatrists, social workers, and community-based organization staff.

### Survey design and administration

An online Survey Monkey^©^ link was sent to eligible participants through their personal email addresses between March to November 2019. An email reminder was sent if the survey was not initiated within three weeks. The survey could be completed in either English or French and had two parts. Part 1 had eight questions on information related to sociodemographic characteristics of participants. Part 2 presented 100 areas of concern within each domain and subdomain that could potentially be a barrier to ART adherence. The domains, subdomains, and the number of areas that were presented to participants are shown in Table 1. The domains, subdomains, and items were obtained from previous work from our group that developed a conceptual framework on perceived barriers to ART from the literature and qualitative semi-structured interviews with people living with HIV.^4,34,35^

**Table 1.** Domains, subdomains, and number of items included in the survey.

Domains	Subdomains	Number of items
My activities (lifestyle factors)	Demands and organisation of daily life	14
	Substance use	2
My thoughts and feelings (cognitive and emotional aspects)	Knowledge	2
	Motivation	3
	Acceptance of HIV	2
	Beliefs	17
	Affect	8
My health (health experience and state)	Bodily signals	4
	Medical signs of HIV and general health	5
	Comorbidity	2
My situation (social and material context)	Relations with others	6
	HIV stigma and privacy concerns	4
	Challenging material circumstances	3
My medication (characteristics of antiretroviral therapy)	Side effects	5
	Instructions	4
	Physical features	4
My care (healthcare services and system)	Patient-provider relationship	5
	HIV clinic issues	4
	Pharmacy issues	5
	Drug cost coverage	1

The participants rated each area on “importance for HIV care” on a four-point Likert scale: no (1), somewhat (2), quite^36^, and very (4). Participants also had the option to add comments. The survey was translated into French by native bilingual translators. Informed consent was administered through the survey link. Ethical approval for the study was obtained from the Research Ethics Board (REB) of the McGill University Health Centre’s Research Institute in Montreal, Quebec, Canada.

### Data analysis

Descriptive statistics, proportion, mean, and standard deviation, were used to describe the respondents. Each area was ranked based on the proportion of respondents: groups (people living with HIV and professionals), countries (Canada/France), and sexes (females/males). An area was deemed to be important for HIV care if it was rated at 3 or 4 (quite or very). The ranks on importance were tested using the Chi-square test separately for people living with HIV and professionals. Significance was set at *p* ≤ 0.05.

## Results

Table 2 shows the response rate and sociodemographic characteristics for people living with HIV and healthcare professionals. The survey was sent out to 50 people living with HIV and 74 healthcare professionals. The response rate in Canada and France, for people living with HIV was 93% (26/28) and 64% (14/22) whereas that for professionals was 84% (32/38) and 67% (24/36). respectively. Two respondents self-identified as trans individuals. One of them also identified as male, and their data were combined with male respondents. Data on the second trans individual is not presented to avoid any risk of disclosure.

**Table 2.** Response rate and sociodemographic characteristics of the respondents: people living with HIV (*n* = 40) and healthcare professionals (*n* = 56).

Variables	Mean (SD) or N (%)
**People living with HIV**	
Survey response (completed/sent)	
Canada	26/28 [93]
France	14/22 [64]
Sex: Female/Male	22 [55]/7 [43]
Language: English/French	16 [40]/24 [60]
Education	
Primary	4 [10]
Secondary	9 [23]
College/CEGEP/technical degree	13 [34]
University	11 [28]
Other	2 [5]
Sexual orientation	
Heterosexual	23 [58]
Homosexual	13 [33]
Bisexual	3 [8]
History of drug use: Yes/No	10 [25]/30 [75]
**Healthcare professionals**	
Survey response (completed/sent)	
Canada	32/38 [84]
France	24/36 [67]
Language: English/French	28 [50]/28 [50]
Gender: Female/Male	38 [68]/18 [32]
Involvement in HIV care (years)	
5–9	28 [51]
10–14	7 [14]
15–19	8 [15]
≥20	12 [20]
Service location	
Hospital	35 [65]
Private clinic	7 [13]
Both	12 [22]

Table 3 presents the areas endorsed as important adherence barriers for HIV care by; groups (people living with HIV and care professionals), countries (Canada and France), and sexes (females and males). The Chi-square test was statistically significant for the important qualifier at *p* ≤ 0.05. Only those areas endorsed as important by over 50% of the respondents within each comparison group are highlighted. A total of 43 of 100 areas were endorsed as important ART adherence barriers. Specifically, people living with HIV endorsed 18 of 43 areas (42%) (all highlighted cells under the people living with HIV category) whereas professionals endorsed 24 of 43 (56%) (all highlighted cells under professionals) across the country and sex. Finally, 15 of 43 (35%) areas were deemed important barriers for consideration in HIV care by both groups, across country and sex. These 15 common areas were: (1) Not having insurance to cover my medication costs or not having enough coverage;^37^ Not having a stable or suitable place to live;^38^ Being tired of taking my medication every day; (4) Being concerned about stigma or discrimination related to HIV; (5) Feeling sad or depressed; (6) Having another health condition to deal with (for example, depression, diabetes or heart disease); (7) Not feeling motivated to take my medication; (8) Having side effects that interfere with my daily activities; (9) Not wanting others to notice that I take this medication; (10) Fearing rejection because of HIV; (11) Having side effects from my medication; (12) An irregular or unpredictable schedule; (13) Forgetting; (14) Feeling my medication is toxic or harmful; (15) Having trouble accepting that I have HIV. These 15 areas belonged to domains and subdomains, which are: My care - drug cost coverage (1 area); My situation - Challenging material circumstances (1 area), HIV stigma and privacy concerns (3 areas); My thoughts and feelings - Affect (2 areas), Motivation (1 area), Beliefs (1 area), Acceptance of HIV (1 area); My health - Comorbidity (1 area), My medication - Side effects (2 areas), My activities - Demands and organisation of daily life (2 areas). Three areas were least endorsed by people living with HIV: (1) My medication schedule conflicting with my sleep pattern,^37^ A change to my daily routine, and^38^ Having other priorities in my life than taking my medication. For the healthcare professionals, the one area that was least endorsed was being too sick or ill.

**Table 3.** Areas endorsed as important by the people living with HIV and healthcare professionals by sex and country.

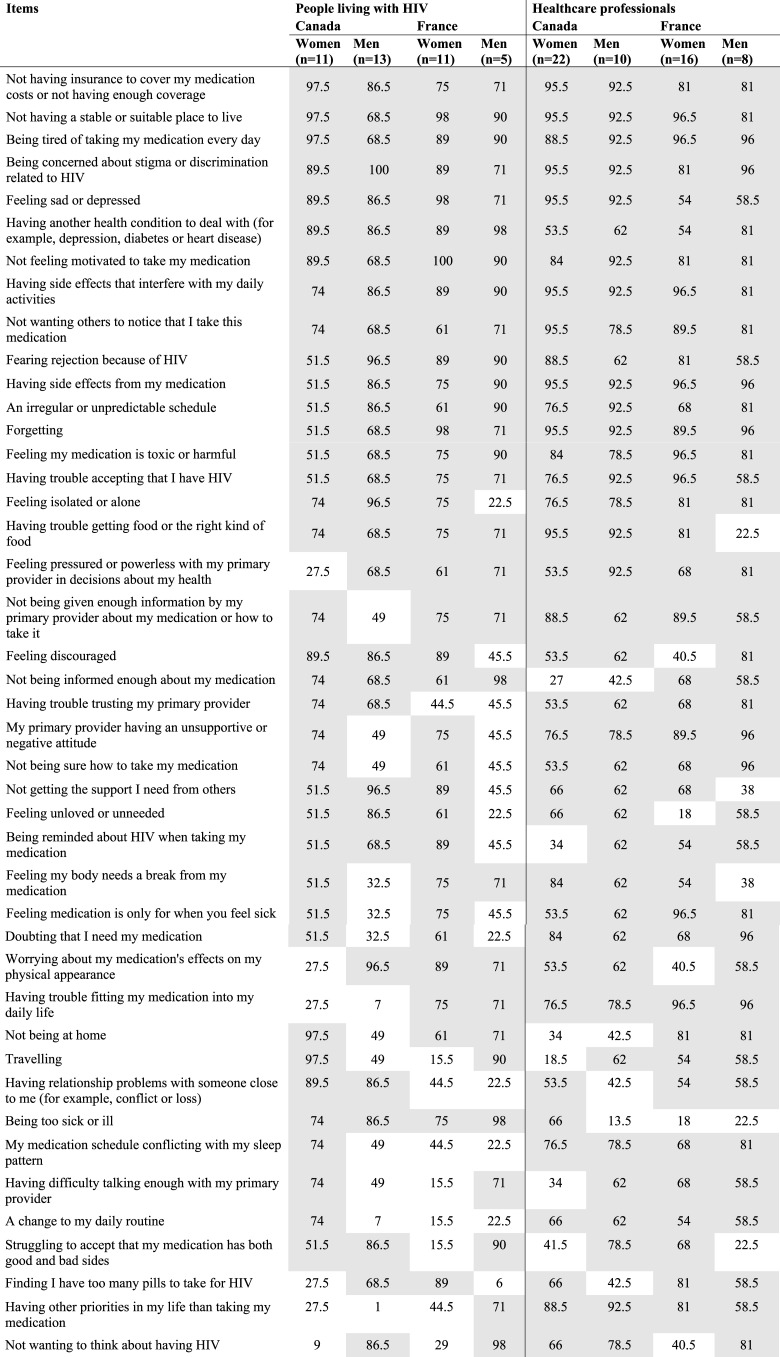

Highlighted cells indicate that the item was endorsed as important by the group.

Table 4 presents the important areas endorsed by the people living with HIV and care professionals and the corresponding subdomains, and domains. All areas in the three subdomains; drug cost coverage (one item), acceptance of HIV (two items), and knowledge (two items), were endorsed as important. The following areas endorsed covered the subdomains: HIV stigma and privacy concerns [3 of 4 items; 75%], challenging material circumstances (2 of 3 items; 67%), relations with others (3 of 4 items; 75%), side-effects (3 of 5 items; 60%), demands and organisation of daily life (8 of 14 items; 57%), and comorbidity (1 of 2 items; 50%). Less than 50% of items from the subdomain in affect, motivation, beliefs, bodily signals, instructions, and medical signs of HIV and general health were endorsed as important barriers to HIV care. None of the areas from the four following subdomains was endorsed by the respondents (see Tables 1 and 4): (1) substance abuse,^37^ physical features,^38^ HIV clinic issues, and (4) pharmacy issues.

**Table 4.** Areas and corresponding subdomains and domains endorsed as important barriers to HIV care by the people living with HIV and healthcare providers.

Domains	Sub-domains	Important areas endorsed
	(Endorsed/administered)^b^	
My care (healthcare services and system)	Drug cost coverage (1/1)	Not having insurance to cover my medication costs or not having enough coverage^a^
My situation (social and material context)	Challenging material circumstances (2/3)	Not having a stable or suitable place to live^a^
		Having trouble getting food or the right kind of food
	HIV stigma and privacy concerns (3/4)	Being concerned about stigma or discrimination related to HIV^a^
		Not wanting others to notice that I take this medication^a^
		Fearing rejection because of HIV^a^
	Relations with others (4/6)	Feeling isolated or alone
		Not getting the support I need from others
		Feeling unloved or unneeded
		Having relationship problems with someone close to me (for example, conflict or loss)
My thoughts and feelings (cognitive and emotional aspects)	Affect (3/8)	Being tired of taking my medication every day^a^
		Feeling sad or depressed^a^
		Feeling discouraged
	Motivation (1/3)	Not feeling motivated to take my medication^a^
	Beliefs (5/17)	Feeling my medication is toxic or harmful^a^
		Feeling medication is only for when you feel sick
		Doubting that I need my medication
		Struggling to accept that my medication has both good and bad sides
		Being reminded about HIV when taking my medication
	Acceptance of HIV (2/2)	Having trouble accepting that I have HIV^a^
		Not wanting to think about having HIV
	Knowledge (2/2)	Not being informed enough about my medication
		Not being sure how to take my medication
My health (health experience and state)	Comorbidity (1/2)	Having another health condition to deal with (for example, depression, diabetes or heart disease)^a^
	Bodily signals (1/4)	Feeling my body needs a break from my medication
	Medical signs of HIV and general health (1/5)	Being too sick or ill
My medication (characteristics of antiretroviral therapy)	Side effects (3/5)	Having side effects that interfere with my daily activities^a^
		Having side effects from my medication^a^
		Worrying about my medication’s effects on my physical appearance
	Instructions (1/4)	Finding I have too many pills to take for HIV
My activities (lifestyle factors)	Demands and organisation of daily life (8/14)	An irregular or unpredictable schedule^a^
		Forgetting^a^
		Having trouble fitting my medication into my daily life
		Not being at home
		Travelling
		My medication schedule conflicting with my sleep pattern
		Having other priorities in my life than taking my medication
		A change to my daily routine
My care (healthcare services and system)	Patient-provider relationship (5/5)	Feeling pressured or powerless with my primary provider in decisions about my health
		Not being given enough information by my primary provider about my medication or how to take it
		Having trouble trusting my primary provider
		My primary provider having an unsupportive or negative attitude
		Having difficulty talking enough with my primary provider

^a^Endorsed by both groups, people living with HIV and healthcare professionals.

^b^Substance abuse, physical features, HIV clinic issues, and pharmacy issues are the four subdomains that were not endorsed.

## Discussion

The overall objective of this study was to identify perceived barriers to ART adherence from the perspective of people living with HIV and healthcare and social service professionals. Of the 100 potential barriers administered by the survey, 43 were endorsed by at least 50% of participants, both people living with HIV healthcare and social service professionals (Table 3).

All groups (people living with HIV and healthcare professionals) across countries (Canada and France) were concordant on the following barriers to HIV adherence: (1) Not having insurance to cover my medication costs or not having enough coverage, (2) Not having a stable or suitable place to live, (3) Being tired of taking my medication every day, (3) Being concerned about stigma or discrimination related to HIV, (4) Feeling sad or depressed, (5) Having another health condition to deal with (for example, depression, diabetes or heart disease), (6) Not feeling motivated to take my medication, (7) Having side effects that interfere with my daily activities, (8) Not wanting others to notice that I take this medication, (9) Fearing rejection because of HIV, (10) Having side effects from my medication, (11) An irregular or unpredictable schedule, (12) Forgetting, (13) Feeling my medication is toxic or harmful, and (14) Having trouble accepting that I have HIV (see Table 3). The fact that these areas were identified as barriers to ART adherence by most of the people in this study necessitates the need to develop strategies to mitigate these barriers, particularly those who are likely to be poor adherent. Some of these barriers are discussed below.

Within the Canadian context, insurance coverage was identified as the most important barrier to ART by all groups except males living with HIV (ranked third). Cost-related non-adherence in Canada for any prescribed drug is estimated to be around 9.6% (95% CI: 8.4%–10.7%).^39^ This translates to around 1 in 10 Canadians incurring out-of-pocket expenses for ART medications. Insufficient insurance coverage is a main predictor of high cost-related non-adherence.^40^ Furthermore, lack of insurance coverage is also shown to be associated with four times greater odds of not filling out a prescription (Odds Ratio (OR) 4.52, 95% CI: 3.29–6.20).^39^ Insurance coverage is worrisome in a country such as Canada where public health coverage covers for all in-hospital costs, but prescription drugs are financed through various public and private insurances and that the coverage differs across provinces. This complex and fragmented drug coverage leaves many people living with HIV vulnerable and prone to non-adherence and highlights the need to foster a more uniform and affordable coverage.

The second important area endorsed as a barrier to ART adherence is stigma and discrimination (ranked second for females living with HIV). This finding is concurrent with the literature. Rintamaki et al. reported a 3.3 times greater likelihood of non-adherence to ART among those who were concerned about HIV stigma compared to those who were less concerned.^41^ Stigma, as a barrier to ART adherence, needs further exploration to identify the sources of stigma, whether structural or intersectional and its mediating mechanisms.^42^ Without fully understanding the interaction between stigma and non-adherence, any strategy to mitigate the barrier is unlikely to be effective. For example, Rao and colleagues demonstrated depressive symptoms as causally mediating the relationship between HIV-related stigma and HIV medication adherence.^28^ A systematic review of healthcare professionals’ attitudes, beliefs, and behaviors towards people living with HIV showed that professionals with limited HIV stigma training were more likely to overtly display stigmatizing behaviors towards their patients.^43^ Several strategies are proposed in the literature to reduce structural stigma and including introspection, cultural awareness, and sensitization workshops.

The third top barrier, according to this survey’s participants, was the lack of a stable or suitable place to live (ranked fourth by males living with HIV). People living in vulnerable households were more likely to be nonadherent, according to a Health and Housing in Transition study that surveyed 596 vulnerable housed individuals and 595 homeless across three Canadian cities.^44^ A similar study showed a negative association between homelessness and ART adherence as well as plasma viral load.^45^ While this barrier may be outside the purview of the healthcare system, regular communication or contact with the healthcare team could be implemented to prevent people who are likely to fall through the cracks. This is not unimaginable in the era of technological advances. For example, short message services (SMS or text message) are effective method to improve adherence to ART (odds ratio (OR) 1.48, 95% credible interval 1.0–2.2).^46^

When ranks are compared among respondents in France, not feeling motivated to take my medication and not having a stable or suitable place to live were the top two barriers endorsed by both people living with HIV and professionals across sex. Incorporating strategies to motivate people living with HIV is shown to improve adherence to ART. Several motivational strategies are effective. Frisher and colleagues proposed an Information-Motivation-Behavioral Skills model to guide development interventions to support and improve adherence to ART.^47^ For instance, motivational interviewing is shown to increase prescribed doses taken compared to the control group.^48^ The hypothesis is that motivational interviewing builds confidence, reduces ambivalence, and increases motivation to adhere to ART. The absence of a suitable place to live is not a country-specific barrier and warrants further exploration.

A systematic review of patient-reported barriers to adherence to ART from 125 studies involving 17,061 adults reported forgetting, being away from home, and change to a daily routine as the most frequently reported barriers.^24^ The current study concurs with these findings but highlights that these barriers are not patient perceptions alone but are also shared by healthcare professionals.

Effective physician-patient communication and problem- solving dialogues are the cornerstone for successful health outcomes.^49^ In HIV care, the Centers for Disease Control and Prevention recommends brief conversations with patients that facilitate opportunities to identify barriers and benefits of ART adherence. For instance, HIV patients could be screened in some 15 areas identified as barriers prior to their clinical visits. Indeed, to avoid the respondent burden, a maximum of 20–25 questions (10–15 min) is recommended,^6^ and not all areas need to be administered at each visit. A study by Laws and colleagues have shown that a patient-physician dialogue improves adherence and that these conversations could be started by addressing the ART adherence barriers endorsed by both people living with HIV and professionals.

Future implications of these results would be to develop a dedicated care team to identify people living with HIV who report barriers to ART adherence. A bidirectional communication platform could be created between the care team and the patient to identify on-going challenges and develop individualized strategies to improve ART adherence.

## Limitations

This study has several limitations that warrant attention. First, the sample of people living with HIV and healthcare and social service professionals from Canada and France may not be representative of the populations. The authors acknowledge this inherent limitation in the survey design. Second, responding to 100 items could have resulted in respondent interest lapses. However, in the early stages of identifying barriers to ART, it is necessary to identify and present issues that cover all aspects of possible barriers from the perspective of the people living with HIV and healthcare professionals. Fourth, the study did not collect information on whether people with HIV and their corresponding healthcare and social service professionals responded to the survey. This information would have helped to understand if some of these barriers are nested within a particular region or sex.

## Conclusions

Our study supports some of the widely reported areas that are potential barriers to adherence to ART. The strength of this study is that some of the perceived barriers to HIV care were shared by both people living with HIV and healthcare professionals. This study also highlighted some novel barriers such as insurance coverage, challenges with material circumstances such as a living place or food, and bodily signals as barriers to ART adherence by people living with HIV and healthcare professionals. Targeted interventions could be developed in some of these common areas to minimize the barriers and support people living with HIV to improve adherence to ART.
